# Experimental Animal Model Systems for Understanding Salivary Secretory Disorders

**DOI:** 10.3390/ijms21228423

**Published:** 2020-11-10

**Authors:** Ji-Youn Kim, Chang-Hyeon An, Jae-Young Kim, Jae-Kwang Jung

**Affiliations:** 1 Department of Dental Hygiene, College of Health Science, Gachon University, Incheon 21936, Korea; hoho6434@gachon.ac.kr; 2 Department of Oral and Maxillofacial Radiology, School of Dentistry, IHBR, Kyungpook National University, 2177, Dalgubeol-daero, Jung-gu, Daegu 41940, Korea; chan@knu.ac.kr; 3 Department of Biochemistry, School of Dentistry, IHBR, Kyungpook National University, 2177, Dalgubeol-daero, Jung-gu, Daegu 41940, Korea; jykim91@knu.ac.kr; 4 Department of Oral Medicine, School of Dentistry, IHBR, Kyungpook National University, 2177, Dalgubeol-daero, Jung-gu, Daegu 41940, Korea

**Keywords:** animal experimental model, salivary dysfunction, salivary gland, therapeutic strategies

## Abstract

Salivary secretory disorders are life-disrupting pathologic conditions with a high prevalence, especially in the geriatric population. Both patients and clinicians frequently feel helpless and get frustrated by the currently available therapeutic strategies, which consist mainly of palliative managements. Accordingly, to unravel the underlying mechanisms and to develop effective and curative strategies, several animal models have been developed and introduced. Experimental findings from these models have contributed to answer biological and biomedical questions. This review aims to provide various methodological considerations used for the examination of pathological fundamentals in salivary disorders using animal models and to summarize the obtained findings. The information provided in this review could provide plausible solutions for overcoming salivary disorders and also suggest purpose-specific experimental animal systems.

## 1. Introduction

### 1.1. Salivary Gland and Saliva

The oral cavity is a unique, complex structure that is involved in many functions, including respiration, deglutition, mastication, and pronunciation, all of which are essential for the quality and quantity of life [1]. These physiological functions are accomplished by the highly coordinated involvement of the teeth, tongue, palate, gingiva, and other oral mucosa components [1]. Saliva is the aqueous secretion that lines the entire surface of the oral cavity and originates from the acinar cells of salivary glands, and is considered to play a central role in maintaining the integrity and functions of oral compartments [2]. Saliva consists of approximately 99% water and a variety of components including electrolytes, glycoprotein, proteins represented by enzymes, immunoglobulins, and antimicrobial factors, which contribute to the diversity of salivary properties and functions for both oral and general health [2,3]. Salivary glands consist of three pairs of major glands (parotids, submandibulars, and sublinguals), which are located outside the mouth, and a number of minor glands located inside the mouth. Major salivary glands contain three main cell types: acinar cells, ductal cells, and myoepithelial cells [4]. In addition, the connective tissue forms a capsule around the gland and extends into it, dividing groups of secretory units and ducts into lobes and lobules. Blood vessels, lymphatic vessels, and nerves are also present within the capsule for supplying the gland [5]. The functional unit of a salivary gland is called the acini. Acini are composed of epithelial secretory serous and mucous cells and are responsible for the production of saliva. The serous or mucous cells along with myoepithelial cells are arranged in an acinus or acini with a roughly spherical or tubular shape and a central lumen [5]. Myoepithelial cells wrap around the acini and intercalated ducts with four to eight processes, rhythmically contracting to squeeze saliva from the acinar units upon stimulation by nerves, through the duct system, and into the oral cavity [4]. Pathological alterations in secretory elements (acinar, duct, myoepithelial, and nerve) can cause salivary secretory disorders, leading to various life-disrupting pathological events [6].

### 1.2. Salivary Secretory Disorders and Animal Models

Salivary secretory disorders can be caused by a wide range of oral and systemic conditions, leading to histocellular disturbances in the salivary gland and, consequently, a quantitative and qualitative decline in saliva [7]. These conditions include the prolonged use of systemic medications, the application of radiation or radioisotope treatment, glandular pathologies such as sialadenitis and sialolithiasis, and systemic diseases, such as diabetes and Sjögren’s syndrome [8]. Although the therapeutic target should be centered on histopathologic impairment the underlying salivary dysfunction, almost all clinical therapies are helpful for only temporary symptomatic relief without recovery of histofunction [8]. Accordingly, a number of experimental animal models have been developed to clarify the detailed mechanisms underlying the initiation, progression, and recovery in salivary pathologies [9,10,11,12,13,14]. These models were created by the application of excessive irradiation, radioisotope, ductal ligation, inflammogens, mechanical injury, medications, and genetic modifications [6,15,16,17,18,19,20]. A radiation and radioisotope model was developed to reproduce the hyposalivation induced by head and neck cancer therapy. The ductal ligation model aimed to mimic the obstructive sialadenitis and sialithiasis-induced sialopathy. A model of inflammation, mechanical injury, and systemic disease/medications was devised to correspond to Sjögren’s syndrome and virus/bacterial sialadenitis, traumatic injury-induced sialopathy, and sialopathic alterations under diabetes, renal disease, hypertension, and/or their therapeutic medications. Although previous studies using various applications of experimental animal models have reported a variety of histocellular findings, the analytical comprehension of previous methodologies and findings could be advantageous for both clinicians and researchers to help facilitate preclinical research for the future development of new therapeutic strategies.

### 1.3. Objectives

The purpose of this article is to provide a methodological summary to improve future experimental protocols and consolidate previous histofunctional findings underlying salivary glandular impairment and recovery. This review focused exclusively on the practical considerations regarding various experimental procedures applied in animal studies.

## 2. Methods

We searched for available studies in English using the PubMed database (up to April 2020) and references for other literature. The search was performed in PubMed repeatedly using the keywords “salivary gland”, “radiation” (or “irradiation”), “ligation” (or “obstruction”), “animal” (or “animal model”), and other related words. Secondary searches were performed among the references cited in the articles initially found. The collected studies were carefully reviewed from the methodological and histomolecular points of view.

## 3. Results

### 3.1. Experimental Animal Models

Animal models were used to study radiation-induced salivary gland dysfunction, radioactive iodine-induced sialadenitis, acute/chronic obstructive sialadenitis, bacterial sialadenitis, sialithiasis, diabetes-induced salivary dysfunction, and Sjögren’s syndrome. Various animal species, including rat, mouse, cats, pigs, monkeys, sheep, and others, have been used as models of salivary secretory disorders [21,22,23]. Previous studies have confirmed that experimental models reproduced similar histopathological alterations in salivary glands, with some variations between species and individuals; moreover, these alterations were closely related to salivary dysfunction [9,23,24]. Table 1 outlines the previously used animal models and their mimicking salivary diseases or disorders in humans.

**Figure 1:**
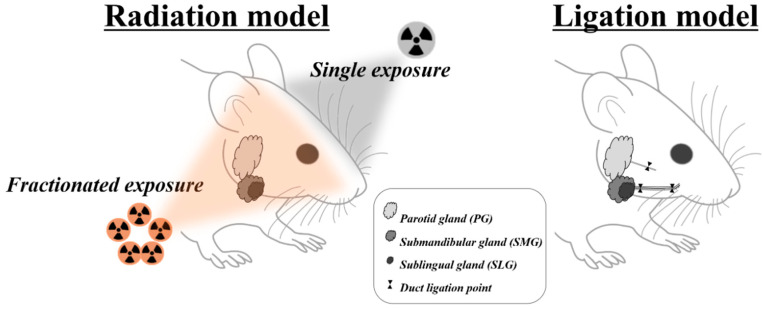
Overview of animal models mimicking radiotherapy- and duct obstruction–induced salivary gland damage.

Dental clinicians are more likely to encounter patients with several sialopathies, such as medication-induced dysfunctions, radiation-induced sialopathy, obstructive or inflammatory siadenitis, and sialithiasis [9,26]. However, upon careful literature review, it was found that animal models for medication-induced dysfunctions were not fully established. Therefore, the remainder of this article will focus solely on the radiation model and ductal obstruction model (Figure 1).

### 3.2. Radiation Model

#### 3.2.1. Experimental protocols

Various malignant tumors occur around the head and neck areas. In 2018, more than 710,000 patients suffered from these tumors, with a corresponding 350,000 deaths worldwide [27]. Although radiotherapy is considered to be one of the main anticancer therapies for head and neck cancers, radiation-induced hyposalivation is also one of most common complications, mainly resulting from the destruction of salivary tissues with their high biological susceptibility to radiation [27,28]. Copious studies using a radiation model have been performed, with a variety of radiation dosages administered over different species [22,28,29,30,31,32]. The irradiation procedure could be divided into single-dose and fractionated irradiation according to its schedules. In contrast to a single high irradiation dose, fractionated irradiation is defined as the application of radiation dosage fractionated into far smaller doses over several weeks [28]. Fractionated irradiations are considered more similar to radiotherapy procedures, with typical time courses of 6 weeks [28,33]. According to the collected literature, mice, rat, minipig, monkey, and rabbit are mainly used for radiation models. Among these, most animal studies include mice and rats. In the mice irradiation model, a single dose of 2–18 Gy or a fractionated dose of 28 Gy (5.6 Gy × 5 days) is performed. In the rat irradiation model, 7.5–20 Gy is used as a single dose, and a total of 20–75 Gy (4, 6, 7, 8, 9, 15 Gy × 5 days) is used as a fractionated dose. The irradiation models using minipig, monkey, and rabbit were produced mainly through a fractionated irradiation of 37.5–70 Gy.

#### 3.2.2. Main Findings

Previous studies have reported that histological alterations such as acinar loss are intimately related to radiation dose [29,34,35,36]. However, it should be considered that irradiation with a single high dose might significantly shorten the life span of the experimental animals [36]. Previous research showed that irradiated salivary glands were characterized histologically with parenchymal loss, acinar atrophy and interstitial fibrosis, duct proliferation, and dilated intercalated and striated duct [6,9,23]. A previous study reported in the rat indicated that a single exposure of 15 Gy induced considerable reductions in the weights of the parotid and submandibular/sublingual glands (by 36% and 24%, respectively) as well as salivary flows of the parotid and submandibular/sublingual glands (by 74% and 46%, respectively) [37]. However, findings regarding the relative radiosensitivity of the salivary glands were conflicting [22,23,37]. Although DNA was considered to be the main critical target of radiodestructive effects, several studies were performed to determine the detailed pathologies of apoptosis in salivary acinar cells. These researchers reported that p53 could be a key molecule in the regulation of radiosensitivity via the probable involvement of DNA damage repair, cell cycle arrest, and apoptosis [28]. Noticeably, it was furthermore revealed that radiation exposure induced no apoptosis in knockout mice with p53^−/−^ as determined by immunohistochemistry of caspase-3 [29]. These findings demonstrated that post-irradiation apoptosis in the salivary gland could be mediated by p53. In addition, other study reported that post-irradiation apoptosis was suppressed by greater than 60% in the parotid glands of PKCδ^−/−^ mice when compared with wild-type mice [38]. This finding indicates that PKCδ is required for the efficient induction of post-radiation apoptosis in salivary epithelial cells.

However, recent studies have indicated that the sharp and persistent decline in salivation flow cannot be fully explained by only the loss of acinar cells after irradiation [28,39,40]. In addition to apoptosis, the down-regulation of acuaporin 5 (AQP5) is considered to be a contributing mechanism of the induction of post-irradiation hyposalivation. It was reported that the expression of AQP5 was down-regulated in the salivary glands with decreased salivation on days three and 30 after irradiation [41]. Other studies have described that the disturbance in the calcium signaling pathway through TRPM2 might underlie the post-irradiation dysfunction in the salivary gland [40,42]. A previous study found that although wild-type mice exhibited prolonged hyposalivation after radiation, the duration of recovery from hypofunction was facilitated in TRPM2^−/−^ mice [43]. Another study revealed that, after irradiation, TRPM2^−/−^ mice displayed only transient loss of STIM1 and Store-operated Ca2+ entry (SOCE) along with transient hyposalivation and, moreover, gene therapy to express STIM1 increased the salivary flow and SOCE [40]. Collectively, recent studies showed that irradiation of the salivary gland was accompanied by extensive deterioration including decreased AQP5 expression, parasympathetic innervation (GFRα2 and AchE expression), regeneration potentials (Shh and Ptch expression), salivary trophic factor levels (brain-derived neurotrophic factor and neurturin), and stem cell expression (Sca-1) [44].

In addition to acinar cells, impairment of nonparenchymal tissues such as the parasympathetic nerve and microvessels was reported as an additional mechanism of radiation injury [6,44,45]. These studies reported that parasympathetic dysfunction and vascular dilation were observed as part of late postradiation effects [6,46]. 

Recent studies have shown a distinctive regeneration pattern in the salivary gland after irradiation, as compared with homeostatic regeneration under normal conditions [6,20]. Under normal conditions, differentiated acinar cells can be maintained and regenerated through self-duplication of acinar progenitors in a lineage-restricted pattern [47,48]. Meanwhile, under normal conditions, the K14-expressing duct cells are known to contribute to the formation and maintenance of ductal structures but not acinar structures [49]. However, it was recently revealed that, after irradiation, the ductal cells as well as acinar cells contribute to the generation and repair of acinar cells [20]. This finding suggests that cellular plasticity would be involved in the restoration of the salivary gland after severe cellular damage, such as irradiation [20] (Table 2).

### 3.3. Ductal Obstruction Model

#### 3.3.1. Experimental Protocols

An animal model with experimental ductal obstruction has been used to mimic sialopathies via obstructive sialadenitis and/or sialithiasis. According to the collected studies, mice, rat, cat, and rabbit were primarily used for the production of ductal obstruction models. Ductal obstruction in rodents was applied mainly in submandibular and less in the parotid gland. This might be because the submandibular gland is the largest and easiest salivary gland to identify grossly [74]. The obstructive period varied considerably from 24 h to one year, which could be suitable for reproducing acute and chronic sialopathies, respectively [21,24,75,76,77]. According to the study design, the removal of obstructers such as silk or clip was often performed to reproduce the repair stage by restoring the ductal passage [75,78,79,80]. Ductal obstruction was mainly achieved by ductal ligation with surgical silk or ductal clipping with an aneurysm clip. More recently, the ductal clipping method was predominantly used because the clipping procedure is a more simple and less traumatic method during both operations of obstruction and, especially, later removal. In addition, to avoid fibrosis of the ducts by minimizing irreversible ductal damage, studies attempted to insert a small plastic tube at the neck or the joint of the clip [11,81]. Although many studies have used the unligated contralateral glands as controls, a previous study used the corresponding gland of an unoperated, naive animal [81]. The possibility was considered that, when the unilateral gland was obstructed, the compensatory hyperplasia would occur in the unligated, contralateral gland [82].

#### 3.3.2. Main Findings

Previous studies revealed a marked decrease (75%) in the weight of the salivary gland after experimental obstruction [17,83,84,85]. The dramatic atrophy could make it more difficult to identify the obstructed gland and then to stitch out the tightly knotted silk without causing any ductal damage [85]. Therefore, the use of a metal clip appears to be more advantageous in terms of convenience and safety. In the past, rats were more frequently used as experimental animals for clipping than mice, probably because of the surgical feasibility, whereas experimental mice were considered to have unique advantages of well-identified genetic backgrounds over other animals. However, since the introduction of the mini-clip, the mouse has been more actively used by overcoming the limitation of size [78,84]. The location of the experimental obstruction is grossly divided into the proximal ductal portion through the extraoral or cervical approach and the distal ductal portion through the intraoral approach [81,85]. On the proximal portion of the salivary duct, the main duct runs together with the parasympathetic nerve and blood vessel by connective sheath. Distal obstruction is usually determined to minimize any compounding effect by possible ligation or clipping of other structures, such as the parasympathetic nerve and the supplying blood vessel [11,79,86] (Figure 1). However, it is not easy to select the distal obstruction in mouse because of the restricted animal size and surgical accessibility. Although it might be possible to avoid the ligation of surrounding blood vessels and nerves under surgical stereoscope, high surgical skill still seems to be needed to perform the surgical procedure in the mouse [85]. 

In both rats and mice, the ductal obstruction was known to cause apoptosis of acinar cells and the proliferation of duct cells [82,87,88]. An excessive increase in the intrasalivary pressure acted as the physical trauma on acinar cells, consequently leading to cellular death, whereas ductal cells remained grossly intact but with ductal dilation [89,90]. A previous study showed that the apoptotic reaction occurred throughout the acinar cells several days after the obstruction, and the apoptotic cells were then phagocytized by the adjacent acinar cells or intraepithelial macrophages [88]. After 7 days, most acinar cells had disappeared, leaving prominent residual ducts [88]. Another study in the rat reported that the rapid, progressive cellular loss of greater than 85% within the acinar tissue accounted for glandular shrinkage within two weeks after ductal obstruction [91]. 

Although multiple mechanisms are involved in the apoptotic response and subsequent secretory dysfunction after ductal obstruction, several mechanisms might be related to the disturbance in membrane receptors and signaling pathway of acinar cells. A previous study revealed up-regulation of the mitogen-activated protein kinases, extracellular signal-regulated receptor kinase 1/2, and p38 during the atrophic and regeneration phases of ductal obstruction/release [92]. Another study showed that P2Y increased about 15-fold three days after obstruction, and this increase returned to the control level by 14 days after removal of the obstruction [93]. The expression level of AQP5 after ligation was also decreased with the localization at the apical membranes of the remained acinar cells [94]. 

In addition, a previous study showed the apoptotic reaction in ductal cells, the marked shortage of intercalated ducts, and the dilation of ductal and acinar lumens [82,95]. It also reported that the observed apoptosis of the capillary endothelial cells was related to the reduction in the capillary bed [82]. Other studies demonstrated that the expression of Bcl-2 was increased in the ductal cells after the ductal obstruction [77].

Previous studies also showed that after ductal obstruction, myoepithelial cells underwent both apoptotic and proliferative reaction, leading primarily to changes in the distribution and morphology rather than rapid disappearance [96,97,98]. Furthermore, it was also shown that myoepithelial cells seldom participated in the regeneration of atrophied glands, despite their proliferation and differentiation [82,98]. 

With regard to the repair process, studies have shown that the histomorphology of the gland returned to almost normal after duct recanalization [24,79,81]. Acinar cell recovery was considered to involve redifferentiation from the remaining cells [79]. A recent study showed that the duct and acinar cell lineages were maintained separately, even after indirect mechanical injury such as obstruction [20]. However, others studies suggested the possible involvement of cellular plasticity between the residual ductal and acinar populations during salivary regeneration [78,95] (Table 3).

## 4. Discussion

The development of a new conceptual therapy for the regeneration of the salivary gland has long been required. Although histocellular information is essential to fully understand the various pathologic conditions of salivary dysfunctions, histological evaluation is not clinically available for salivary glands in most patients. This is because, although frequent sialopathies underlying salivary dysfunctions are not a life-threatening condition, biopsy is an invasive procedure, possibly causing complications such as infection, poor wound healing, fistula, and scar formation. Therefore, it is insufficient to establish the histocellular knowledge through biopsy in humans [9]. Recently, animal models mimicking diseases/conditions have been developed to study the functional alteration of salivary glands [6,20]. The experimental application of an animal model could allow for the examination of histomorphological and physiological information as well as facilitate the development of therapeutic strategies by reproducing various salivary pathologic conditions. However, there are some considerations to note in current animal models of salivary disorders. Radiation experiments in animals have been used as a good model for identifying the sequential reaction of the salivary gland after radiotherapy. Most studies using the animal model have reported a decrease in salivation rate and gland weight and loss of acinar cells [29,34,35,36]. However, some studies have reported conflicting results, showing little inflammation, cell apoptosis, and acinar cell loss after irradiation [57]. The main difference in radiation animal studies is the amount of radiation required for a significant loss of salivary gland functionality. For a more precise analysis, it is necessary to prove the optimal irradiation-induced salivary gland disorder after radiotherapy. In addition to most of the irradiated animal models were produced in a manner that is dissimilar to the actual human radiation therapy. Recently relatively large animals, such as monkeys and mini-pigs, have been used to mimic salivary gland dysfunction of humans [22,23,32]. The animals can be utilized as a more feasible biological model for studying salivary gland dysfunction because the salivary glands of valuable large animals are anatomically and physiologically similar to human glands [22,23,32]. However, researchers should be aware that physiological differences in salivary function among species can cause bias in animal modeling as shown in Table 4. Furthermore, in the ductal obstruction model, the optimal obstruction period has not been determined because of the diverse atrophic or apoptotic alterations according to the given periods, although most studies maintained the ductal obstruction for one or two weeks [17,95]. These variations are partly explained by the technical diversity in ligation [95,116]. Some researchers have indicated that the application of single ductal ligation with a surgical suture might fail to obtain complete blockage of duct, thereby escaping some acinar cells from apoptotic conditions even after prolonged obstruction [88]. Furthermore, an early study reported that, despite the application of the same ductal obstructive procedure, the severity of the atrophic reaction varied widely [116]. In addition, Sjögren’s syndrome is a systemic autoimmune disease that affects the salivary gland. Recent studies have highlighted the impact of the depletion of stem cells as a factor contributing to the loss of gland regeneration [118]. However, the development of treatments for the salivary gland disorder of Sjögren’s syndrome has been hindered by insufficient animal models that can completely reproduce the human condition.

Recently, researchers have attempted to implement novel therapeutic strategies, such as stem cell or gene therapy, delivery of bioactive compound, and bioengineering approaches for the functional regeneration of salivary glands [119]. Appropriate therapeutic strategies should be applied depending on the cause of the salivary gland disorder. Salivary gland disorders in the radiation model are characterized by cell damage and fibrosis [6]. The Sjögren’s syndrome model is characterized by the destruction of inflammatory tissue and consequent depletion of stem cells in the salivary gland [118]. With aging, salivary glands are shown to undergo histomorphological changes and functional alterations, such as the reduction of salivation and increase in apoptotic epithelial cells [120]. Therefore, treatment of salivary gland disorders should be individualized for each patient according to the causes of cell damage and pattern of tissue alteration. The most appropriate and effective treatment should be provided for the functional regeneration of the salivary gland, such as stem cell transplantation, bioactive compound or gene delivery to damaged cells, or bioengineering approach. However, the therapeutic options have been mostly applicated in a radiation-induced xerostomia model. Very little research has been performed in the animal models of ligation-induced salivary gland damage or Sjögren’s syndrome. In addition, many studies have used an irradiated submandibular gland to examine the functional regeneration of the salivary gland [119]. Potential therapeutic strategies should be applied and analyzed in various animal models of salivary gland dysfunction. An accurate understanding of the molecular mechanism involved the in functional regeneration of salivary glands will be needed to restore salivary function lost as a consequence of side effects of radiotherapy, various diseases, or mechanical injuries affecting the salivary glands. 

Among experimental animals, murine models are regarded as excellent candidate due to not only their histomorphological similarities with humans, but also experimental conveniences [121,122]. However, questions remain regarding whether the results of studies in murine models could produce the same results in humans. Since murine salivary glands used in animal experiments showed a similar, but different, anatomical physiology compared with humans (Table 5), it is important to consider that no single animal model system has covered all aspects of the pathogenesis and clinical features of each pathological condition. For this reason, various protocols using animal models have been applied to recapitulate clinical pathologies as much as possible. In particular, establishing and applying an animal model that more precisely resembles and mimics the human pathologic conditions is fundamental. These proper animal model systems would be an opportune chance to promote our understanding of destructive and reparative mechanisms under physiological and pathological conditions. Furthermore, existing and future animal model systems will provide the fundamental evidence to justify progressive experiments as preclinical steps. In the future, accumulated findings will facilitate the ability of clinicians to provide permanent and not merely palliative treatment.

## Figures and Tables

**Table 1:** Summary of the experimental animal models mimicking sialopathies.

Type of Animal Model	Experimental Method	Aimed Pathologic Diseases/Disorders
Radiation model	Irradiation using experimental radiation equipment [9,20]	Radiation-induced sialopathy/sialadenitis
Radioisotope model	Administration of radioiodine [13,19]	Radioactive iodine-induced sialadenitis
Ductal obstruction (ligation) model	Obstruction of salivary duct using surgical suture or aneurysm clip [11,17]	Obstructive sialadenitis, sialithiasis-induced sialopathy
Inflammation model	Application of autoimmune disease animal models or inflammogens [14,15]	Sjögren’s syndrome, bacterial sialadenitis
Mechanical injury	Direct induction of tissue damage mainly using biopsy punch [10,16]	Traumatic injury–induced sialopathy
Systemic diseases/medications	Application of systemic disease animal models and/or their therapeutic medications [12,18,25]	Sialopathic alterations under diabetes, renal disease, hypertension, and/or their therapeutic medications

**Table 2:** Irradiation protocols used in previous studies.

Animal	Target Gland	Dose (Total)	Fraction No.	F/U Period	Ref.
Mice	PG	2, 5 Gy	Single	1, 2, 3, 4, 30 days	[29]
		5 Gy	Single	5, 10, 15, 30 min, 1, 2, 4 h 30, 60 days 4, 5, 7, 30 days 8, 24 h 1, 2, 3 days	[50] [51] [52] [53] [54]
	SMG	10 Gy	Single (5 Gy per side)	3, 7, 14, 30 days	[55]
		12 Gy	Single	4, 8, 12 weeks	[56]
		13 Gy/28 Gy	Single/fraction (5.6 Gy × 5 days)	48, 72 h, 2, 8 weeks	[57]
		15 Gy	Single	8, 24 h, 4 weeks 90 days 3, 7, 28 days 8 weeks (once a week) 1, 3, 10, 30 days	[58] [59] [60] [61] [40]
		18 Gy	Single	30, 60, 90 days	[62]
	SLG	10 Gy	Single	1, 3, 7, 14 days	[63]
	PG, SMG	5 Gy	Single	1, 2, 3, 4, 5, 30, 60, 90 days	[64]
		15 Gy	Single	4 h, 8 weeks 30, 65 days	[45] [65]
	PG, SMG, SLG	5 Gy	Single	9, 30, 60, 90 days	[66]
		15 Gy	Single	10, 30, 60, 120 days	[43]
Rat	PG	15 Gy	Single	7 days	[30]
		20 Gy	Single	1 days	[67]
		30, 40 Gy	Fraction (6 or 8 Gy × 5 days)	180 days	[35]
	SMG	7.5 Gy	Single	14 days	[68]
		15 Gy	Single	3, 30 days	[41]
		18 Gy	Single	4, 7, 28, and 56 days 8, 16, and 24 weeks	[69] [70]
		20 Gy	Single	7 days	[71]
		75 Gy	Fraction (15 Gy × 5) every second week	6 weeks	[72]
	PG, SMG	2.5, 5, 7.5, 10, 15 Gy	Single	3, 6, 9, 12 months	[36]
		15 Gy	Single	2 months	[37]
		20, 35, 45 Gy	Fraction (4 or 7 or 9 Gy × 5 days)	2, 4, 10, 180 days	[34]
	PG, SMG, SLG	15 Gy	Single	3, 70 days	[73]
		18 Gy	Single	2, 6, 8, or 12 weeks	[44]
Minipig	PG	37.5 Gy	Fraction (7.5 Gy × 5 days)	12 weeks	[32]
	PG, SMG	70 Gy	Fraction (2 Gy × 35) daily, except weekends	1 month	[23]
Monkey	PG, SMG	50, 55 Gy, CHART	50 Gy in 20 fraction 55 Gy in 25 fraction CHART	16 weeks (fortnightly)	[22]
Rabbits	PG, SMG	10, 20, 30, 40 Gy	Fraction (2 Gy × 5, 10, 15, 20 days)	immediately	[31]

PG, parotid gland; SMG, submandibular gland; SLG, sublingual gland; CHART, continuous hyperfractionated accelerated radiotherapy.

**Table 3:** Obstruction protocols in previous studies.

Animal	Target Gland	Obstruction Protocol			Duration		Ref.
		Material	Method	Control	Obstruction	Deobstruction	
Mice	SMG (unilateral)	6–0 Ethicon suture		Normal	3, 5, 7 days	−	[99]
	SMG (unilateral)	Metal Sugita titanium aneurysm clip		Normal	7 days	0, 1, 3, 7, 14 days	[78]
	SMG (unilateral)	Silk thread		Normal	8 weeks	−	[76]
	SMG (unilateral)	Surgical sutures		Contralateral	0, 1, 3, 6, 12, 24 h, 1, 3, 6 days	−	[94]
	SMG (unilateral)	Sugita titanium aneurysm clip		Contralateral	2 months	1 week, 1, 2 months	[84]
	SMG (unilateral)	Surgical sutures		Contralateral	7 days	28 day	[85]
Rat	PG (bilateral)	3–0 silk ligature		Sham	7 days	7, 30 days	[92]
	PG	Suture		Normal	1, 7, 15, 21, 30, 60 days	−	[100]
	PG (unilateral)	Clip		Sham	14 days	2, 8, 14, 21, 25, 28 days	[101]
	PG (unilateral)	Liu et al. 1998			2 weeks	1, 2 weeks	[91]
	PG (unilateral)	Ligaclips, double clipped		Sham	7 days	0, 1, 2, 3, 4, 5, 7, 10, 14 days	[79]
	PG (unilateral)	Metal clips, double clipped		Normal	7 days	0, 1, 3, 5, 7, 10, 12, 14, 17, 21 days	[102]
	PG (unilateral)	4–0 silk, double ligated		Normal	12,18 h/1, 2, 3, 4, 5, 7 days/2,3,4,8,12,24 weeks	−	[82]
	SMG (unilateral)	Surgical sutures	7 mm distal to the gland hilum	Contralateral	3 days	3, 7, 14, 21 days	[93]
	SMG (unilateral)	Metal clip		Contralateral	1 day	3 days	[24]
	SMG (unilateral)	Metal clip		Contralateral	4 weeks	8 weeks	[83]
	SMG (unilateral)	Metal microclip	5 mm posterior to the ductal orifice	Contralateral	1 day	−	[75]
	SMG+SLG	Metal clip		Normal	2 weeks	3 days	[81]
	SMG+SLG	Metal clip		Normal	2 weeks	3, 5, 7 days, 8 weeks	[103]
	SMG	3–0/8–0 silk sutures	Midportion/the orifice of the duct	Normal	1, 3, 5, 7 days	−	[104]
	SMG (unilateral)	−		Normal	1, 3, 7, 14, 21 days	−	[105]
	SMG (unilateral)	Surgical vascular ligation clip	5 mm distal to the glandular porta	Contralateral	1, 3, 7 days	1, 2 weeks	[106]
	SMG	8–0 suture		Sham	2, 3, 4 weeks	−	[17]
	SMG (unilateral)	Metal microclip	5 mm posterior to the ductal orifice	Contralateral	1, 4, 8 weeks	8, 16, 24 weeks	[11]
	SMG (bilateral)	Metal clip	Near the hilum	Normal	7 days	0, 1, 3, 5, 7, 11 days	[80]
	SMG (bilateral)	Metal clip		Normal	7 days	0,3,7,14 days	[107]
	SMG (unilateral)	Metal clip, double ligation		Normal	1,2, 3, 4, 5, 7, (10), 14, (21), 28 days	−	[88,96]
	SMG(unilateral)	Metal clip, double ligation	Near the hilum of the gland	Normal	7 days	0, 1, 2, 3, 4, 5, 7, 10, 14 days	[108,109]
	SMG (unilateral)	Ligaclips, double ligation	Near the hilum of the gland	Normal	1, 3, 5, 10, 14 days	−	[110,111]
	SMG, SLG (bilateral)	−	At a distance of 2 mm from the organs	Sham	2 weeks	−	[112]
	SMG(+SLG) (unilateral)	Metal microclip	less than 5 mm posterior to the ductal orifice/less than 5 mm anterior to the hilum of the gland	Contralateral	1, 2, 7, 14, 21 days	−	[86]
	SMG (+SLG) (unilateral)	Metal microclip	5 mm posterior to the ductal orifice	Normal	1, 3, 5, 7, 9, 14 days	−	[113]
	SMG, SLG (unilateral)	Metal clips, double ligation	Near the hilum of the gland	Normal	1, 3, 5, 7, 10, 14, 28 days	−	[114]
	SLG (unilateral)	Metal clip, double ligation	Near the hilum of the SLG	Normal	1, 3, 5, 7, 10, 14, 28 days		[97]
	SLG (unilateral)	Metal clip/ligaclips, double ligation	Near the hilum of the SLG	Normal	7 days	0, 1, 2, 3, 4, 5, 7, 10, 14 days	[98,115]
Cat	PG (unilateral)	3–0 silk sutures		Contralateral	From 1 to 365 days	−	[21]
	PG, SMG, SLG (unilateral)	3/0 braided silk		Contralateral	From 1 to 365 days	−	[116]
Rabbit	PG (bilateral)	3–0 silk suture		Sham	1, 7, 14, 30, 60 days	−	[95]
	SMG	6–0 nylon thread	At 5 mm behind the orifice of the duct		2, 4, 8 weeks	−	[117]

PG, parotid gland; SMG, submandibular gland; SLG, sublingual gland; CHART, continuous hyperfractionated accelerated radiotherapy.

**Table 4:** Histopathological change in animal model system of salivary gland dysfunction.

Animal Model	Histopathological Analysis
Radiation	Mouse/Rat Acinar loss and atrophy, interstitial fibrosis, duct proliferation, and dilated intercalated and striated duct ^‡^ [29,34,35,36] Little inflammation, cell apoptosis, and acinar cell loss after irradiation * [57]
	Mini-pig Marked acinar atrophy, fibrosis, parenchymal loss, duct proliferation, and dilated intercalated and striated duct in the irradiated parotid and submandibular gland [23,32] Apparent decline in stimulated salivary flow by 81% and gland size by about 50% [23]
	Monkey Severe atrophy and fibrosis in parotid and submandibular glands [22] Drastic loss of acinar cells by over 95% [22]
Ligation	Mouse/Rat Apoptosis of acinar cells and the proliferation of duct cells [82,87,88]
	Rabbit Marked acinar atrophy, reduction in the number of acinar cells by apoptosis in parotid gland, Slower progression of sialopathies relative to rat [95] Acinar atrophy, infiltration of inflammatory cells, interstitial fibrosis, and duct expansion in submandibular gland [117]
	Cat At 4 days after ligation of parotid gland, acini change including vacuolation, disintegration, extravasation, apoptosis, phagy, and a reduction in number and size of secretory granules [21] Existence of residual acinar cells of parotid even after 1 year [21]

^‡^^,^* It has been reported conflicting results.

**Table 5:** Comparative anatomical and histological findings of major salivary glands.

	Human	Rodent
Parotid	Main composition with serous acini Prominent striated and intercalated ducts Prominent intralobular adipose tissue	Main composition with serous acini Prominent striated and intercalated ducts Less prominent intralobular adipose tissue *
	Location-anteroinferior area of ear Size-1st largest	Location-posteroinferior area of ear * Size-2nd largest *
Submandi-bular	Mixed composition with both serous and mucous acini Marked demilunes Well-developed striated and intercalated ducts	Mixed composition with predominant serous acini * Less/no marked demilunes * Well-developed striated and intercalated ducts Prominent granular convoluted tubule producing various growth factors *
	Location-submandibular area Size-2nd largest	Location-ventral cervical area * Size-1st largest *
Sublingual	Mostly comprised of mucous acini	Mostly comprised of mucous acini
	Location-sublingual area Size-smallest	Location-ventral cervical area * Size-smallest

* The asterisk denotes the anatomical and histological difference.

## Abbreviations

PG	Parotid gland
SMG	Submandibular gland
SLG	Sublingual gland
AQP5	Acuaporin 5
SOCE	Store-operated Ca2+ entry
